# Investigation of the Fracture Characteristics of a Cement Mortar Slab under Impact Loading Based on the CDEM

**DOI:** 10.3390/ma16010207

**Published:** 2022-12-26

**Authors:** Qunlei Zhang, Decai Wang, Jinchao Yue, Chun Feng, Ruifu Yuan

**Affiliations:** 1 School of Civil Engineering and Communication, North China University of Water Resources and Electric Power, Zhengzhou 450045, China; 2 School of Water Conservancy and Environment, Zhengzhou University, Zhengzhou 450001, China; 3 Institute of Mechanics, Chinese Academy of Sciences, Beijing 100190, China; 4 School of Energy Science and Engineering, Henan Polytechnic University, Jiaozuo 454003, China

**Keywords:** cement mortar slab, impact cracking, CDEM, hammerhead shape, impact velocity

## Abstract

For brittle and quasi-brittle materials such as rock and concrete, the impact-resistance characteristics of the corresponding engineering structures are key to successful application under complex service environments. Modeling of concrete-like slab fractures under impact loading is helpful to analyze the failure mechanism of an engineering structure. In this paper, simulation models of impact tests of a cement mortar slab were developed, and a continuum–discontinuum element method (CDEM) was used for dynamic analysis. Concretely, the cracking simulations of a mortar slab when considering the hammer shape and impact velocity were conducted, and the impact process and failure results of the slab structure were analyzed. The results showed that the top fracture area of the mortar slab was significantly smaller than that of slab bottom under impact loadings of the drop hammer. The impact velocity was an important factor that affected the mortar slab’s cracking. With the increase in the initial impact velocity, the effective fracture area of the slab structure increased significantly; the impact force and rupture degree of the mortar slab also showed a linear growth trend. The shapes of the impact hammerhead also had a significant effect on the crack model of the mortar slab. The effective fracture zones of slab structures were close under circular and square hammers, while the effective fracture zone was significantly larger under a rectangular hammer impact. The peak value (45.5 KN) of the impact force under a circular hammer was significantly smaller than the peak value (48.7 KN) of the impact force under the rectangular hammer. When considering the influence of the stress concentration of the impact hammerhead, the maximum impact stress of the rectangular hammer was 147.3 MPa, which was significantly greater than that of the circular hammer impact (maximum stress of 87.5 MPa). This may have meant that the slab structures were prone to a directional rupture that mainly propagated along the long axis of the rectangular hammerhead. This impact mode is therefore more suitable for rehabilitation and reconstruction projects of slab structures.

## 1. Introduction

**Figure 1:**
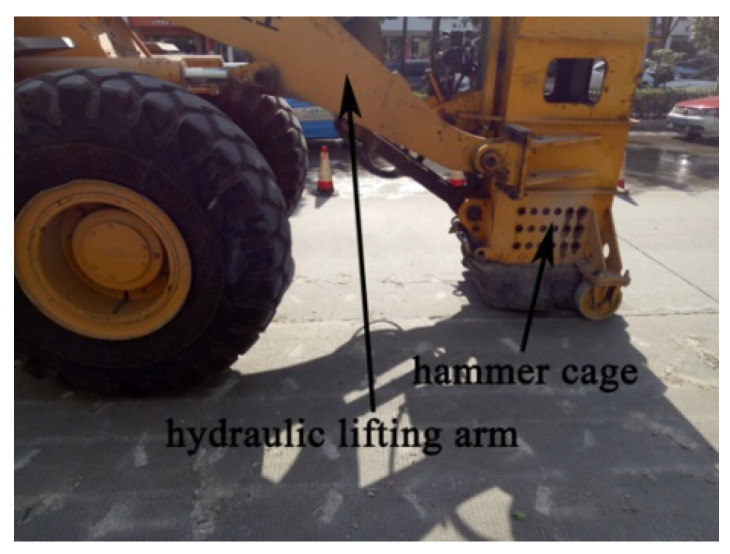
Micro-crack homogenization crushing of pavement structure [2].

Slab structures composed of rock and concrete materials are widely used in roads, airports, and various municipal and hydraulic projects due to their convenient construction and strong bearing capacity. However, many serious defects have appeared in the engineering structures with an increase in the service life. For example, due to the increase in heavy-duty vehicles, a series of defects such as broken slabs, dislocations, cracks, spalling, and potholes have appeared on concrete pavement. Currently, various techniques for rehabilitation, demolition, and reconstruction have been proposed [1]. Micro-cracking homogenization technology makes full use of the residual strength of the original pavement structure through a low-speed impact treatment, which can realize the green recycling and rapid construction of concrete pavement, as shown in Figure 1 [2]. Currently, a method to determine the specific impact mode to effectively fracture the concrete pavement is an important factor, and the understanding of the fracturing mechanism of concrete slab structures is key to the successful application of various technologies. Therefore, investigations of the impact fracture of slab structures will provide guidance for the renovation and reconstruction of old pavement structures.

As for the effect of the impact velocity, it has an obvious penetration effect when the velocity is greater than 500 m/s [3]. As for an impact velocity that is less than 50 m/s, elastic and plastic deformation of the collision are mainly presented [4]. For low-speed collisions, impact tests with a drop hammer are commonly used to investigate the damage effect of explosion or impact on engineering structures. A large number of experimental studies have been conducted on the impact resistance of concrete materials and structures under a low-speed collision. Chen et al. [5] investigated the high-mass, low-velocity impact behavior of concrete members including beams and slabs. Under a three-point bending configuration based on the free-fall of an instrumented impact device, Dey et al. [6] investigated the impact response of fiber-reinforced aerated concrete. Using a drop-weight impact test machine, Yoo et al. [7] conducted impact testing to estimate the impact resistance of concrete slabs strengthened with fiber-reinforced polymer sheets. Zhang et al. [8,9] investigated the flexural impact responses of steel-fiber-reinforced concrete to further analyze the effect of the hammer weight and drop height on the impact performance. The effect of support condition variation on impact behaviors of concrete slabs was investigated through impact loading tests [10]. Radnic et al. [11] investigated the impact behavior of two-way conventional reinforced concrete slabs. Sakthivel et al. [12] developed reinforced cementitious composites and studied their impact performance through a low-velocity repeated-drop-weight impact test. Yahaghi et al. [13] studied the impact resistance and crack characteristic of lightweight concrete reinforced with polypropylene fiber. Elavarasi et al. [14] investigated the response of thin concrete slabs with and without reinforcement under low velocity impacts. As for the effect of the loading rate, dry concrete had an obvious strain-rate-strengthening effect, and the compressive strength and flexural tensile strength were positively correlated with the loading rate [15]. Mei et al. found that the failure modes of concrete beams and columns under an impact load were similar and showed shear failure under high-strain-rate conditions [16]. Gu considered concrete slabs that showed flexural–tensile failure under a quasi-static load local punching shear failure under an impact load [17].

Due to the complexity of the impact-cracking process of concrete structures, successive observations of the crack-propagation process are difficult, and limited data on the impact cracking of concrete structures can be obtained in experiments. In combination with impact experiments, simulation has become an effective means to further study the fracture mechanism of concrete materials and structures due to the development of computer technology. Nia et al. [18] conducted impact tests based on the testing procedure recommended by ACI Committee 544, and the simulation results of concrete materials were compared with experimental testing data. Gopinath et al. [19] conducted experimental and numerical investigations on concrete panels under low-velocity impact loading. Othman et al. [20,21,22] not only conducted an experimental investigation to understand the effect of steel reinforcement distribution on the dynamic response of concrete plates under impact load, but also established a three-dimensional finite element model to verify with the test results. Xiao et al. [23,24] conducted impact tests to study the effects of loading rates and other parameters on concrete slab performances and proposed a two-degrees-of-freedom dynamic model to predict the responses of reinforced concrete slabs subjected to low-velocity impacts. Zhao et al. [25] conducted the impact-response testing of concrete panels through drop-hammer tests and simulations. As in the above simulation studies, concrete and rock failures can be analyzed via FEM, which can accurately calculate the stress and deformation of materials and structure but cannot simulate the fracturing and crushing processes well.

Based on the continuum–discontinuum element method (CDEM), the influence of hammer elastic deformation on concrete pavement failure can be considered through the continuum element method, the cracking process of pavement structures can be well simulated via the discontinuum element method, and the interaction between the drop hammer and the pavement structure can be realized via the contact element [26]. Therefore, the CDEM is a more appropriate method to simulate the impact-cracking process of concrete-like materials and structures. However, under lower impact loading, the cracking simulation of concrete-like slabs while considering the impact-head shape and impact velocity using the CDEM has not been reported in the literature. In this study, the continuum–discontinuum element method (CDEM) was introduced, and simulation models of the drop-hammer impact test are developed. Then, the coupled calculation of the continuum element method and the discontinuum element method was used to simulate the impact-cracking process of a cement mortar slab. Further, the influences of different hammerhead shapes and impact velocities on the fracture mechanism of a mortar slab structure were analyzed.

## 2. Continuum–Discontinuum Element Method

**Figure 2:**
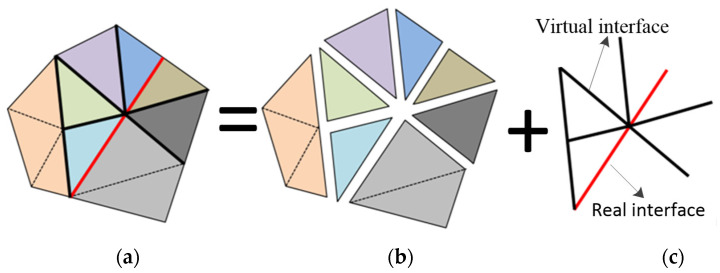
Model schematic of continuum–discontinuum element method: (**a**) numerical model; (**b**) block model; (**c**) interfaced model.

The model schematic of the continuum–discontinuum element method (CDEM) is shown in Figure 2. The CDEM couples finite element calculations with discrete element calculations. It conducts the finite element calculations inside the block elements and conducts the discrete element calculations at the element boundaries [26].

For every nodal mass of a block element, the equilibrium equations can be expressed in the following matrix form in an element (Equations (1) and (2)):

(1) $$\left[M\right]\left\{\ddot{u}\right\}+\left[K\right]\left\{u\right\}+\left[C\right]\left\{\dot{u}\right\}={\left\{F\right\}}_{ext}$$

(2) $${\left\{F\right\}}_{ext}={\left\{F\right\}}_{s}+{\left\{F\right\}}_{t}$$

where [M] denotes the nodal mass matrix; [C] is the damping matrix; [K] denotes the stiffness matrix; {u} denotes the displacement vector; and ${\left\{F\right\}}_{ext}$ denotes the vector of external forces, which include the contact force ${\left\{F\right\}}_{s}$ and the external loading force ${\left\{F\right\}}_{t}$.

In this method, the acceleration is iterated by the central difference scheme, and the velocity is iterated by the unilateral difference scheme. The schemes can be written as following Equations (3)–(6):

(3) $${\left\{\ddot{u}\right\}}_{i}=\frac{{\left\{u\right\}}_{i+1}-2{\left\{u\right\}}_{i}+{\left\{u\right\}}_{i-1}}{{\left(\Delta t\right)}^{2}}$$

(4) $${\left\{\dot{u}\right\}}_{i}=\left({\left\{u\right\}}_{i+1}-{\left\{u\right\}}_{i}\right)/\Delta t$$

(5) $${\left\{\dot{u}\right\}}_{i+1}={\left\{\dot{u}\right\}}_{i}+{\left\{\ddot{u}\right\}}_{i}\Delta t$$

(6) $${\left\{u\right\}}_{i+1}={\left\{u\right\}}_{i}+{\left\{\dot{u}\right\}}_{i+1}\Delta t$$

where ${\left\{\ddot{u}\right\}}_{i}$, ${\left\{\dot{u}\right\}}_{i}$, and ${\left\{u\right\}}_{i}$ represent the acceleration, velocity, and node displacement at the i-th time step, respectively; ${\left\{\dot{u}\right\}}_{i+1}\;$ represents the node velocity at the i+1 time step; and ${\left\{u\right\}}_{i+1}\;$ represents the node displacement at the i+1 time step.

The stress and deformation of an elastic block are calculated using the following Equations (7)–(10):

(7) $$\left[\Delta \varepsilon_{i}\right]=\left[B_{i}\right]\cdot \left[\Delta u\right]$$

(8) $$\left[\Delta \sigma_{i}\right]=\left[D\right]\cdot \left[\Delta \varepsilon_{i}\right]$$

(9) $$\left[\sigma_{i}\right]=\left[\sigma_{i}{}^{0}\right]+\left[\Delta \sigma_{i}\right]$$

(10) $$\left[F_{b}\right]=\overset{N}{\underset{i=1}{\sum }}\left[B_{i}{}^{T}\right]\Delta \left[\sigma_{i}\right]\cdot \omega_{i}\cdot J_{i}$$

where i is the element Gaussian point, $\left[\Delta \varepsilon_{i}\right]$ is the incremental strain vector, $\left[B_{i}\right]$ is the strain matrix, [Δu]  is the incremental displacement vector of the node, $\left[\Delta \sigma_{i}\right]$ is the incremental stress vector, [D] is the element elastic matrix, $\left[\sigma_{i}\right]$ is the total stress at the current step, $\left[\sigma_{i}{}^{0}\right]$ is the total stress at the previous step, $F_{b}$ is the node internal force vector, *N* is the Gaussian point number, $\omega_{i}$ is the integral coefficient, and $J_{i}$ is the Jacobian determinant value.

In the discontinuous element method, the normal and tangential forces of the contact element are calculated using Equation (11):

(11) $$F_{i}\left(t_{1}\right)=F_{i}\left(t_{0}\right)-k_{i}\cdot A_{c}\cdot \Delta du_{i},\;i=1,2,3$$

where $F_{1}$ denotes the normal force of the contact element; $F_{2}\;$ and $F_{3}$ denote the tangential forces of the contact element, respectively; $t_{0}$ and $t_{1}$ denote the current time step and the next time step, respectively; $k_{1}$ denotes the normal stiffness; $k_{2}\;$ and $k_{3}$ denote the tangential stiffnesses, respectively; $A_{c}\;$ is the contact element area; $\Delta du_{1}$ represents the relative displacements in the normal direction; and $\Delta du_{2}$ and $\Delta du_{3}$ represent the relative displacements in the tangential directions, respectively.

To characterize and simulate the fracture process of brittle and quasi-brittle structures composed of rock and concrete materials, the damage failure criterion of the maximum tensile stress and Mohr–Coulomb are applied to the element interface of the fracture area, which considers the tensile fracture energy and shear fracture energy of brittle and quasi-brittle materials.

The damage fracture criterion of the maximum tensile stress while considering the tensile fracture energy following Equation (12):

(12) $$\begin{array}{l} \mathrm{if}-F_{n}(t_{1})\geq \sigma_{t}(t_{0})A_{c}\\ \mathrm{then}F_{n}(t_{1})=-\sigma_{t}(t_{0})A_{c}\\ \sigma_{t}(t_{1})=-{\left(\sigma_{t0}\right)}^{2}\times \Delta u_{n}/(2G_{ft})+\sigma_{t0} \end{array}\}$$

where *σ_t_*_0_*, σ(t*_0_*),* and *σ(t*_1_*)* are the tensile strength of the interface spring element in the initial time step, the current time step, and the next time step, respectively; Δ*u_n_* stands for the relative displacements in the normal directions; and *G_ft_* represents the tensile fracture energy (Pa × m).

The Mohr–Coulomb damage fracture criterion while considering shear fracture energy following Equation (13):

(13) $$\begin{array}{l} \mathrm{if}F_{s}(t_{1})\geq F_{n}(t_{1})\times \tan\phi +c(t_{0})A_{c}\\ \mathrm{then}F_{s}(t_{1})=F_{n}(t_{1})\times \tan\phi +c(t_{0})A_{c}\\ c(t_{1})=-{\left(c_{0}\right)}^{2}\times \Delta u_{s}/\left(2G_{fs}\right)+c_{0} \end{array}\}$$

where *ϕ* denotes the internal friction angle of contact element; *c*_0_, *c(t*_0_*),* and *c(t*_1_*)* stand for the cohesion strength in the initial time step, the current time step, and the next time step, respectively; Δ*u_s_* stands for the relative displacements in the tangential directions; and *G_fs_* represents the shear fracture energy (Pa × m).

## 3. Numerical Model, Mechanical Parameters, and Simulation Scheme

### 3.1. Numerical Models of Impact Test

**Figure 3:**
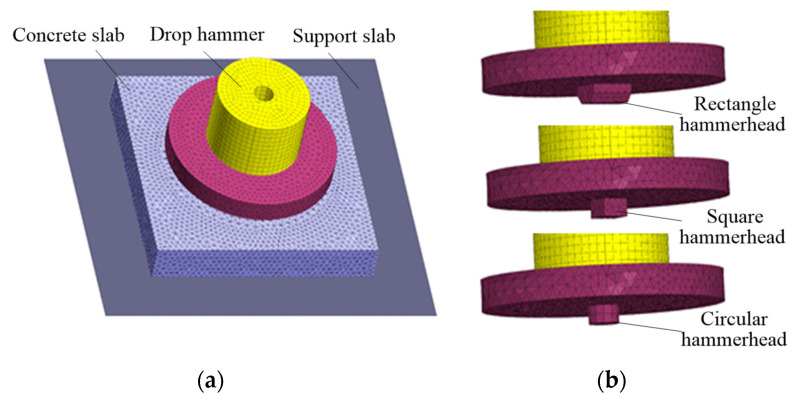
Numerical models of mortar slab impact test: (**a**) impact system model; (**b**) hammer model.

Based on the impact test [2,27], the models of the drop hammer, bearing plate, cement mortar slab, and rigid support slab were established separately, the hammerhead shapes were circular, square, and rectangular as shown in Figure 3. The size of the mortar slab was 400 mm × 400 mm × 60 mm, the size of the impact contact area of the rectangular hammerhead was 20 mm × 54 mm, the size of the square hammerhead was 33 mm × 33 mm, and the section radius of the square hammerhead was 18.6 mm.

### 3.2. Mechanical Parameters in Simulation

In the simulation, the drop hammer and bearing load plate were analyzed accurately by using the continuous element method and adopting the elastic constitutive law. The calculation parameters included the density, elastic modulus, and Poisson’s ratio. The discontinuous element method was used to analyze the impact cracking of the cement mortar slab. The damage fracture criteria of the maximum tensile stress and Mohr–Coulomb were applied in the element interface of the mortar slab. The calculation parameters included the normal contact stiffness, tangential contact stiffness, cohesion, internal friction angle, and tensile strength. The fracture energy parameter was 100 Pa/m. A normal contact element was used to deal with the contact between the impact hammer, mortar slab, and rigid support slab. While considering the impact rebound between the hammer and mortar slab, the strength parameters of the impact contact interface and the support interface were set to zero, and only the normal contact stiffness was required as input. For the continuous–discontinuous calculation of the mortar slab cracking, the block element parameters of the mortar slab, drop hammer, and bearing plate are shown in Table 1. The interface element parameters of the entire impact system are shown in Table 2.

### 3.3. Simulation and Analysis Schemes

In the simulation of the impact cracking of the cement mortar slab, the drop hammer contained the impact kinetic energy by providing an initial impact velocity, which equivalently replaced the free-drop process of the impact hammer in the experiment. Based on the contact element and dynamic explicit algorithm of the CDEM, the simulation of impact-cracking process of the entire system could be achieved by the drop hammer with the impact kinetic energy. During the impact-cracking process of the mortar slab, the time-history curve of the impact-reaction force was obtained by accumulating the contact force between the support slab and the mortar slab. The cracking simulations under the impact loadings of the circular, square, and rectangular hammers were carried out to investigate the effect of the hammer shape on the cracking mechanism of the mortar slab. Moreover, the cracking processes of the mortar slab under the impact velocities of 3 m/s, 4 m/s, and 5 m/s were also simulated to study the effect of the impact velocities on the cracking mechanism. Further, the impact-cracking mechanisms of the mortar slab were investigated by analyzing the relationship between the impact force and the rupture degree.

## 4. Simulation Results and Analysis

### 4.1. Effect of Hammer Shapes on Cracking Mechanism

To study the cracking mechanism of a cement mortar slab under the impact loading of differently shaped hammerheads, we conducted cracking simulations using the circular, square, and rectangular hammerheads. The slab size was 400 mm × 400 mm × 60 mm, and the impact velocity of the drop hammer was 4 m/s.

#### 4.1.1. Analysis of Impact Fracture Forms on the Cement Mortar Slab

**Figure 4:**
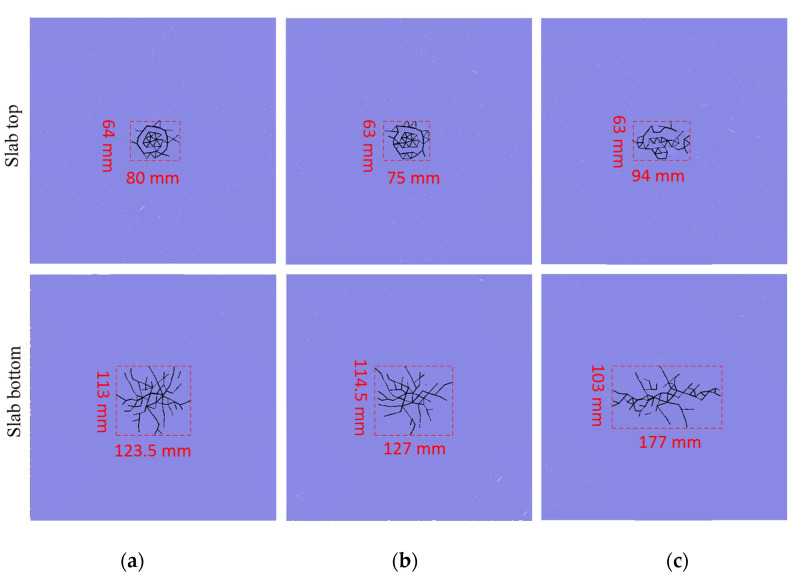
Crack distribution on mortar slab under differently shaped hammerheads: (**a**) circular hammer impact; (**b**) square hammer impact; (**c**) rectangular hammer impact.

Under the impact loadings of differently shaped hammers, the fracture mode of the mortar slab was analyzed via the crack distribution on the slab’s top and bottom as shown in Figure 4.

As shown in Figure 4, the degree of the cement mortar slab fracture was relatively low under an impact loading of 4 m/s, and the impact fracture occurred only in the slab’s middle. The slab’s top was crushed in the impact contact area; however, the larger range of the slab’s bottom mainly showed crack propagation due to the impact bending. The crack model on the slab top showed the circular, square, and rectangular distributions that corresponded to the shapes of the impact hammerhead. Specifically, the effective fracture area of the mortar slab’s top under the impact of the circular-section hammer was 5120 mm^2^, the fracture area of the slab’s top under the square-section hammer was 4725 mm^2^, and the fracture area of the slab’s top under the rectangular hammer was 5922 mm^2^. The slab’s bottom produced symmetrical dispersion cracks under the circular and square hammer heads, while the slab’s bottom cracks under the rectangular hammerhead mainly propagated along the longitudinal direction of the impact head. The effective fracture area of the slab’s bottom under the circular-section hammer was 13,956 mm^2^. The fracture area of the slab’s bottom under the square-section hammer was 14,478 mm^2^. The slab’s bottom fracture area under the rectangular cross hammer was 18,231 mm^2^.

Overall, the failure of the cement mortar slab under the impact loading of 4 m/s was relatively smaller, and the failure occurred only in the slab’s middle. The effective fracture areas of the slab’s top and bottom were relatively close under the circular and square hammerheads; however, the fracture area under the rectangular-section hammer was significantly greater than those of the circular and square hammers. The top fracture areas of the slab were significantly smaller than those of the slab’s bottom under differently shaped hammerheads.

#### 4.1.2. Impact-Stress Analysis of Hammerheads

**Figure 5:**
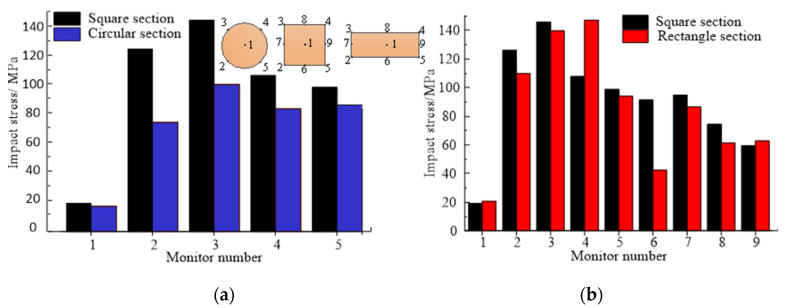
Stress distributions at different positions of impact section: (**a**) comparison of square and circular hammers; (**b**) comparison of square and rectangular hammers.

When the initial impact velocity was the same, the stress concentration caused by the differently shaped hammers affected the initiation position and expansion route of the slab’s top cracks, which led to the generation of different fracture forms on the concrete slab. To further study the influence of the hammerhead shapes on the cracking mechanism, the stress distribution during the impact process was obtained by monitoring the contact element force at different locations of the impact contact surface. Under the impact loadings of the differently shaped hammerheads, the effect of the stress concentration on the impact cracking was determined as shown in Figure 5.

As shown in Figure 5, when the initial impact velocity and the impact contact area of the hammerhead were constant, the impact stress at the hammer section’s center was significantly less than that of the section boundary, and the hammer stress at the midpoint of the section boundary line was significantly less than that at the impact section corner. Specifically, the center stress of the circular hammer section (15.3 MPa) was smaller than that at the section boundary (average of 78.9 MPa). The corner stress of the square hammer section (average of 119 MPa) was greater than that at the boundary line midpoint of the hammer section (average of 80 MPa), which was greater than that of the hammer section’s middle (average of 19.1 MPa). The section corner stress of the rectangular hammer (average of 121 MPa) was significantly greater than those of the section edge (average of 63.3 MPa). The impact stress at the short edge midpoint of the rectangular section (average of 74.6 MPa) was significantly greater than that at the long edge’s midpoint (average of 52 MPa). The minimum stress at the section midpoint of the rectangular hammer was 21 MPa. The maximum stress of the rectangular hammer (147.3 MPa) was larger than that of the square hammer (145.5 MPa), which was larger than that of the circular hammer (87.5 MPa). This showed that in the impact process of the mortar slab, the stress-concentration degree of the rectangular hammer was greater than that of the square hammer, which in turn was significantly greater than that of the circular hammer.

#### 4.1.3. Impact Reaction and Rupture Degree Analysis

**Figure 6:**
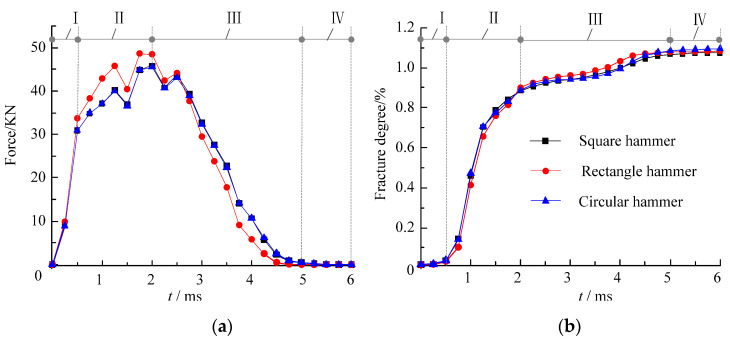
Impact-force and rupture-degree curves under different hammer impacts: (**a**) impact-force curves; (**b**) rupture-degree curves.

As shown in Section 4.1.1, the impact fracture area on the cement mortar slab’s bottom was significantly larger than that of slab’s top, and the fracture characteristics of the slab’s bottom were also different under the different hammer shapes. To further study the influence of the hammer shape on the cracking mechanism, the time-history curves of the impact-reaction force between the mortar slab and the supporting plate were compared and the rupture degrees of the mortar slab were also analyzed (Figure 6).

As shown in Figure 6a, the impact process of the cement mortar slab could be divided into the fast-loading stage Ⅰ, the slow-loading stage Ⅱ, the unloading stage Ⅲ, and the impact-termination stage Ⅳ. At the same time, it can be seen in Figure 6b that the time-history curves of the rupture degree were divided into the initial micro-cracking stage Ⅰ, the rapid-cracking stage Ⅱ, the slow-cracking stage Ⅲ, and the cracking-termination stage Ⅳ. Under the circular, square, and rectangular hammerhead impacts at 4 m/s, the impact process lasted for about 5 ms. Conversely, in the 0–0.5 ms fast-loading stage, the impact-reaction force rapidly increased to 30 KN, but the rupture-degree curve of the mortar slab only slowly increased to 0.05%. In the slow-loading phase of 0.5–2 ms, the impact-reaction force nonlinearly increased to a peak (45 KN) at a lower rate, but the rupture-degree curves showed a rapid growth trend: the rupture degree rapidly increased from 0.05% to 0.9%. In the unloading stage of 2–5 ms, the impact reaction decreased gradually from the peak to 0; meanwhile, the rupture-degree curves of the cement mortar slab showed a slow growth trend, and the rupture degree slowly increased from 0.9% to 1.05%. In the impact-termination stage of 5–6 ms, the impact-reaction force remained 0 and the corresponding rupture degree did not increase.

When comparing Figure 6a,b, under the 4 m/s impact of the square and circular hammers, the time-history curves of the impact force and rupture degree were basically the same. However, the impact-force and rupture-degree curves under the rectangular hammer impact were significantly different from those of the square and circular hammers. Specifically, in the rapid-loading stage of 0–0.5 ms, the curves of the impact force and rupture degree under the different hammer shapes were basically same. In the slow-loading stage of 0.5–2 ms, the magnitude and change rate of the impact force under the rectangular hammer impact were significantly greater than those under the circular and square hammers. However, at this stage, the rupture degree of the mortar slab under the rectangular hammer was slightly smaller than that under the circular and square hammers. In the unloading stage of 2–5 ms, the magnitude and change rate of the impact force under the rectangular hammer were smaller than those of the circular and square hammers, while the change rate of the impact force under the rectangular hammer impact was larger than that of the circular and square hammers. Moreover, in the unloading stage of the rectangular hammer impact, the rupture degree of the mortar slab was generally greater than that of the circular and square hammers.

**Figure 7:**
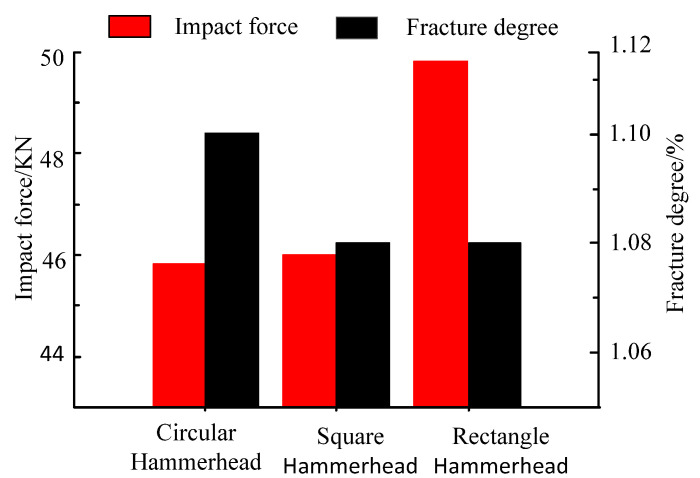
Comparison of impact forces and rupture degrees under different hammer impacts.

To further analyze the influence of the hammer shape on the cracking mechanism of the mortar slab, the peak impact force and final rupture degree under the differently shaped hammers shown in Figure 6 were compared and analyzed as shown in Figure 7.

As shown in Figure 7, the maximum impact force (45.5 KN) of the mortar slab under the circular hammer impact was slightly less than that of the square hammer impact (45.9 KN), which in turn was significantly less than that of the rectangular hammer impact (48.7 KN). However, the ultimate rupture degree of the mortar slab under the circular hammer (1.096%) was significantly greater than that of the rectangular hammer impact (1.083%), which also was greater than that of the square hammer impact (1.075%). This indicated that the impact reaction of the support plate and the rupture degree of the mortar slab showed an opposite variation trend.

### 4.2. Effect of Impact Speed on Cracking Mechanism

To comprehensively investigate the cracking mechanism of the cement mortar slab, we conducted cracking simulations of the mortar slab under the rectangular hammerhead impact. The initial impact velocities of the drop hammer were 3 m/s, 4 m/s, and 5 m/s. The mortar slab’s size was also 400 mm × 400 mm × 60 mm.

#### 4.2.1. Analysis of Impact Fracture Forms on the Cement Mortar Slab

**Figure 8:**
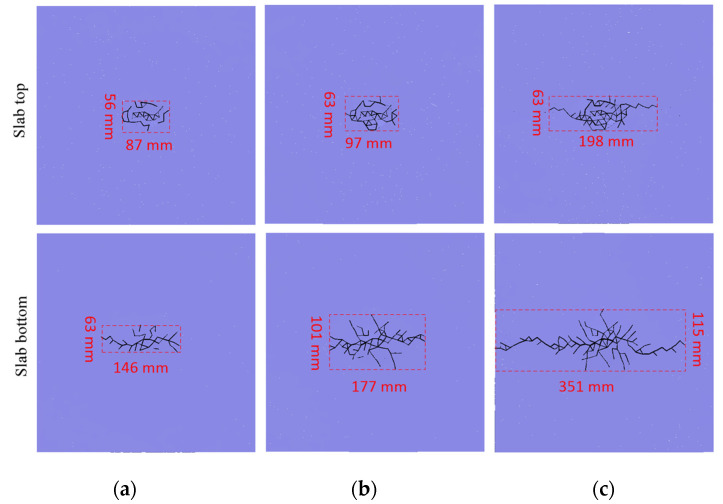
Crack distribution on the slab’s top and bottom under different impact velocities: (**a**) 3 m/s impact; (**b**) 4 m/s impact; (**c**) 5 m/s impact.

To study the influence of the impact velocity on mortar slab fracture, the slab fracture modes under the impact loadings of different velocities were analyzed via the crack distribution pattern as shown in Figure 8.

As shown in Figure 8, under the impact loadings of different initial velocities, the top of the mortar slab was crushed in the impact contact area. However, the slab’s bottom produced cracks that mainly propagated along the longitudinal direction of the rectangular hammerhead. Moreover, the crushing degree of the slab’s top became more severe with an increase in the impact velocity, and the propagation length of the slab’s bottom crack also obviously increased. Under the impact loading with a low initial velocity (3 m/s), the main cracks on the slab’s bottom propagated along the long axis of the rectangular hammerhead. Under the impact loading at the initial velocity of 4 m/s, the main crack length on the slab’s bottom did not increase significantly, but multiple branch cracks on the slab’s bottom were generated along the short-axis direction of the rectangular hammer. Under the impact loading of a 5 m/s initial velocity, the lengths of the main crack and branch crack on the slab’s bottom increased significantly compared with those of the 4 m/s impact. To be more specific, under the impact of the rectangular hammer at the initial velocity of 3 m/s, the effective fracture area on the mortar slab’s top was 4872 mm^2^; the slab’s top fracture area under a 4 m/s impact was 6111 mm^2^; and the top fracture area for the 5 m/s impact was 12,474 mm^2^. Under the impact of the rectangular hammer at 3 m/s, the effective fracture area on the slab’s bottom was 9198 mm^2^; the slab’s bottom fracture area for the 4 m/s impact was 17,877 mm^2^; and the bottom fracture area for the 5 m/s impact was 40,365 mm^2^.

Overall, the effective fracture zone of the mortar slab’s top was relatively close under the impact of the lower initial velocity, but the effective fracture zone of the slab’s top increased significantly under the impact of the larger initial velocity (5 m/s). Moreover, the effective fracture area of the slab’s bottom showed a significantly increasing trend with the increase in the impact velocity.

#### 4.2.2. Impact Reaction and Rupture Degree Analysis of Cement Mortar Slab

**Figure 9:**
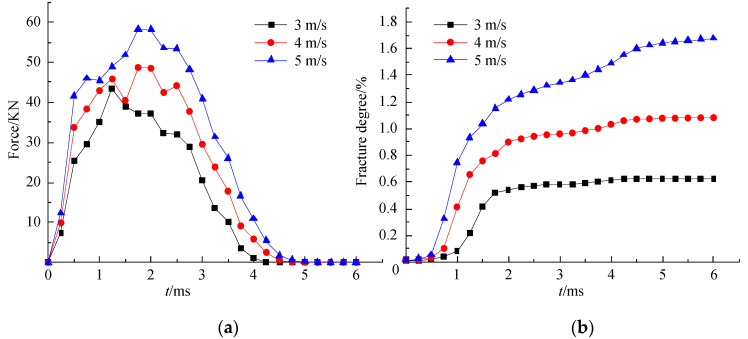
Curves of the impact force and rupture degree under different impact velocities: (**a**) impact-force curves; (**b**) rupture-degree curves.

To further study the effects of the different impact velocities on the cracking of the mortar slab, the impact-force and rupture-degree curves were also compared and analyzed as shown in Figure 9. The impact processes of the mortar slab at different impact velocities lasted for about 3.75–4.5 ms. The impact-reaction force increased rapidly in the 0–0.5 ms fast-loading stage, but the rupture degree of the mortar slab increased slowly to a lower value. In the slow-loading phase of 0.5–2 ms, the impact reaction increased to a peak at a lower rate, but the rupture degree showed a rapid growth trend. In addition, the impact reaction decreased gradually from the peak to 0 in the unloading stage of 2–4.5 ms. The rupture degree showed a significant increase under the impact loading of 5 m/s, and the rupture degree variation of the 4 m/s impact was relatively slow; however, the rupture degree was basically unchanged under the 3 m/s impact. At the impact-termination stage of 4.5–6 ms, the impact-reaction force remained 0, and the corresponding rupture degree of the mortar slab did not increase. The final rupture degree of the 5 m/s impact was obviously greater than that of the 4 m/s impact, which in turn was obviously greater than that of the 3 m/s impact.

When comparing Figure 9a,b, as the impact velocity increased, the impact duration also increased, as did the ultimate rupture degree of the mortar slab. At the same time, as the impact velocity increased, the increase rate of the impact reaction during the loading stage was larger, and the growth rate of the corresponding rupture degree was also larger. In the stage of impact unloading, although the attenuation rate of the impact force and the growth rate of the rupture degree show an increasing trend with the increase in the impact velocity, the rupture degree increased obviously only when the impact speed was higher.

**Figure 10:**
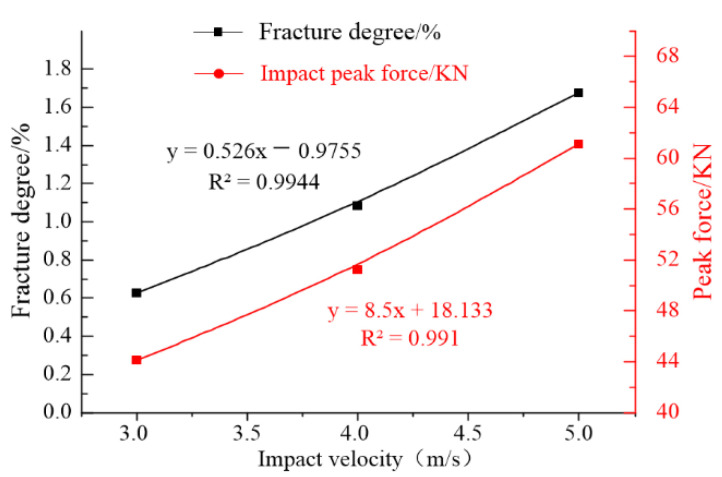
Comparison of the impact force and rupture degree.

To further analyze the influence of the impact velocity, the peak values of the impact force and final rupture degree shown in Figure 9 were fitted and analyzed as shown in Figure 10.

As shown in Figure 10, with an increase in the initial impact velocity of the drop hammer, the maximum impact force on the mortar slab showed a linear growth law; the linear fitting degree R^2^ was 0.991. The final rupture degree of the mortar slab also increased linearly as the initial impact velocity increased; the linear fitting degree R^2^ was 0.9944.

## 5. Discussion

The impact velocity is an important factor in the cracking of concrete-like materials and structures; when the impact velocity is larger, the initial impact energy and the impact force on a cement mortar slab are higher, so the overall rupture degree of the mortar slab is also larger. Moreover, as the impact speed is higher, the change rate of the impact force is larger in the loading and unloading stages, so mortar slabs will fracture not only in the impact-loading stage but also in the impact-unloading stage. Generally, the impact force and overall rupture degree increase with an increase in the impact velocity.

When the initial impact velocity was the same, the stress-concentration degree and stress distribution caused by the differently shaped hammer sections were different, which affected the crack-propagation path, rupture degree, and impact force. Because the impact reaction of the circular and square hammers focused on the slab’s middle, the crushing degree of the mortar slab was larger, and the impact force transmitted to the slab’s bottom was also larger for the rectangular hammerhead’s impact. Therefore, the impact reactions and rupture degrees under the circular and square hammerheads were similar; however, the impact reaction under the rectangular hammerhead’s impact was larger than those of the circular and square hammerheads.

Because the stress-concentration degree and stress-distribution difference of the rectangular hammer were larger than those of the circular and square hammerheads. This corresponded to the section characteristics of the rectangular hammer, and the compression–shear-fracture zone and tension-fracture zone were easier to develop along the long axis. The mortar slab generated the entire fracture under the rectangular hammerhead impact, while the dispersion cracks and local crushing occurred in the slab’s middle under the circular and square hammerheads. Therefore, the rupture degree of the mortar slab under the rectangular hammerhead was lower than those of the circular and square hammerheads.

Further, the specific impact-cracking mode could effectively fracture the concrete material and structure under artificially contorted impact loading; therefore, the rectangular hammer’s impact can be more suitable for application in the renovation of old pavement structures.

## 6. Conclusions

In this paper, the cracking simulations of a cement mortar slab under low-velocity impact were carried out via the continuum–discontinuum element method (CDEM). Then, the influences of the impact velocity and the hammerhead shape on the mortar slab’s cracking were discussed by comprehensively analyzing the slab’s crack distribution, fracture-area size, rupture degree, hammerhead-impact stress, and impact-reaction force of the supporting plate. The following conclusions were drawn:

1. The shapes of the impact hammerheads had a significant effect on the crack model of the mortar slab. The crack model of the slab’s top showed a circular, square, or rectangular distribution that corresponded to the impact-head shapes. The mortar slab’s bottom produced symmetrical dispersion cracks under the circular and square hammerheads. However, the impact cracks mainly propagated along the longitudinal direction of the rectangular hammer. Under the differently shaped hammerheads, the effective fracture area of the mortar slab’s top was significantly smaller than that of the slab’s bottom; under the circular and square hammers, the sizes and shapes of the effective fracture zones on slab’s top and bottom were close, while the effective fracture zone of the rectangular hammer’s impact was significantly greater than those under the circular- and square-section hammers.
2. Under the same initial velocity and different hammerhead shapes, the impact reactions and rupture degrees of the mortar slab showed an opposite trend. The ability to transmit the impact force to the slab’s bottom decreased as the rupture degree increased, so the impact reaction generated by the supporting plate was also smaller. The peak value (45.5 KN) of the impact force under the circular hammer was slightly smaller than the peak value (45.9 KN) of the impact force under the square hammer, which in turn was significantly smaller than the peak value (48.7 KN) of the impact force under the rectangular hammer. However, the final value (1.096%) of the rupture degree under the circular hammer was significantly larger than that under the rectangular hammer (1.083%), which in turn was larger than that under the square hammer (1.075%). The impact velocity was also an important factor that affected the mortar slab cracking. With an increase in the initial impact velocity, the effective fracture area of the mortar slab increased significantly. The maximum impact force showed a linear growth trend and a linear fitting degree R^2^ of 0.991; the final rupture degree also increased linearly and showed a linear fitting degree R^2^ of 0.9944.
3. When the impact velocities and impact areas of the hammerheads were constant, the stress concentration (maximum stress 147.3 MPa) caused by the rectangular hammer’s impact was greater than that (145.5 MPa) of the square hammer’s impact, which in turn was significantly greater than that (87.5 MPa) of the circular hammer’s impact. Therefore, the rupture area, rupture degree, crack form, and impact force of the mortar slab under the rectangular hammer’s impact were significantly different from those of the circular and square hammers. Moreover, corresponding to the section characteristics of the rectangular hammer, the mortar slab was prone to directional rupture; therefore, the rectangular hammer should be more suitable for reconstruction projects involving mortar slab structures.

## 7. Outlook

Slab structures composed of rock and concrete materials are widely used in road, airport, and various municipal and hydraulic engineering. To ensure their normal use, this investigation aimed to provide a theoretical reference for understanding the generation and treatment mechanisms of pavement defects. The interactive relationships of a mortar slab structure and impact drop hammers were discussed, the impact-fracturing processes of the mortar slab under different conditions were simulated, and the crack propagation and failure degree were analyzed. To verify the investigation results based the continuum–discontinuum element method (CDEM), corresponding impact tests will be carried out in the future.

## Figures and Tables

**Table 1:** Constitutive parameters of block elements.

	Density/kg × m^−3^	Elastic Modulus/GPa	Poisson’s Ratio
Hammer	7850	200	0.25
Cement mortar	2400	28	0.2

**Table 2:** Constitutive parameters of contact element.

	Normal Stiffness/Pa × m^−1^	Tangential Stiffness/Pa × m^−1^	Cohesion/MPa	Internal Friction Angle/°	Tensile Strength/MPa
Concrete interface	2 × 10^11^	1 × 10^11^	6	54.9	5
Impact interface	2 × 10^11^	0	0	54.9	0
Support interface	2 × 10^10^	0	0	54.9	0

## Data Availability

Not applicable.
